# CD95/Fas stoichiometry in future precision medicine

**DOI:** 10.1038/s41418-025-01493-9

**Published:** 2025-04-15

**Authors:** Mauricio Sica, Murielle Roussel, Patrick Legembre

**Affiliations:** 1 CONICET, Instituto Balseiro (UNCuyo), Departamento de Física Médica (GAANS-CNEA), Bariloche Atomic Center, Av. Bustillo 9500, Bariloche, Río Negro Argentina; 2 UMR CNRS 7276, INSERM U1262, CRIBL, Université de Limoges, 2, Rue Marcland, Limoges, France; 3 Clinical Hematology and Cellular Therapy Department, CHU Dupuytren, Limoges, France

**Keywords:** Immunological disorders, Molecular modelling

## Abstract

CD95, also known as Fas, belongs to the tumor necrosis factor (TNF) receptor superfamily. The main biological function of this receptor is to orchestrate and control the immune response since mutations in CD95 or deregulation of its downstream signaling pathways lead to auto-immunity and inflammation. Interestingly, more than twenty years ago, pioneer studies highlighted that like TNFR1, TRAILR1 or CD40, CD95 pre-associates at the plasma membrane in a ligand-independent fashion. This self-association occurs through a domain designated pre-ligand assembly domain or PLAD. Although the disruption of this pre-association prevents CD95 signaling, no drugs targeting this region have been generated because many questions remain on the stoichiometry and conformation of this receptor. Despite more than 40.000 publications, no crystal structure of CD95 alone or in combination with its ligand, CD95L, exists. Based on other TNFR members, we herein discuss the predicted conformation of CD95 at the plasma membrane and how these putative structures might account for the induction of the cell signaling pathways.

## Facts

- Metalloprotease-cleaved CD95L (sCD95L) induces non-apoptotic signals
- sCD95L is involved in the progression of auto-immune disorders
- CD95 aggregation relies on its pre-ligand assembly domain (PLAD) and transmembrane domain
- PLAD represents a therapeutic target to block CD95 signals

## Open Questions

- Does plasma membrane CD95 self-association result in parallel or anti-parallel dimers, or can both forms co-exist?
- What is the interface of PLAD CD95?
- Is the PLAD a molecular target to inhibit CD95 signaling?
- How does CD95 signal in a CD95L-independent fashion?

## TNF superfamily

To maintain a robust and controlled immune response against infections and cancer cells, immune and non-immune cells must communicate, a process that occurs through a complex cytokine network. Ligands of the tumor necrosis factor (TNF) superfamily (TNFSF) and their receptors belonging to the TNF receptor (TNFR) superfamily (TNFRSF) contribute to this process by modulating the development and homeostasis of both innate and adaptative immune systems. Twenty-nine TNFRs have been described and all are type I transmembrane proteins, with the exception of BCMA, TACI, BAFFR and XEDAR, which are type III transmembrane proteins [1]. In addition, OPG and DcR3 do not possess a transmembrane domain and are secreted TNFRs [2]. OPG binds RANKL and TRAIL [1, 3, 4], while DcR3 interacts with CD95L [5], TL1A [6] and LIGHT [2]. TNFRSF members share structural features and mechanisms of action. The key feature of these TNFRs is the presence of cysteine-rich domains (CRDs), which are stabilized by cysteine disulfide bonds providing a ladder structure to the receptor [7]. TNFRs possess between one to four CRDs. Interestingly, TNFR1 [8], CD95 [9], TRAILR1 [8] and CD40 [8] self-associate in a ligand-independent fashion through their N-terminal region designated pre-ligand assembly domain (PLAD), which does not contribute to ligand interaction.

## CD95 structure

CD95 (also known as Fas or APO1) is a 335 amino acid long type I transmembrane receptor that belongs to the TNFRSF [10]. Ubiquitously expressed in the human body, this receptor is instrumental in tumor surveillance and immune homeostasis, as evidenced by clinical symptoms in human patients affected by CD95 mutations, who exhibit an auto-immune disorder designated auto-immune lymphoproliferative syndrome (ALPS) type Ia [11]. CD95 belongs to the so-called “death receptor” subset of the TNFRSF which also includes TNFR1, CD95, DR3, TRAILR1, TRAILR2, DR6, NGFR and EDAR [12]. Although the interaction with its cognate ligand, CD95L (FasL or CD178), can implement a caspase-dependent apoptotic signal [13–15], this receptor also exerts multiple nonapoptotic functions [16–21] and many of them are tumor promoting (reviewed in [22, 23]).

**Fig. 1:**
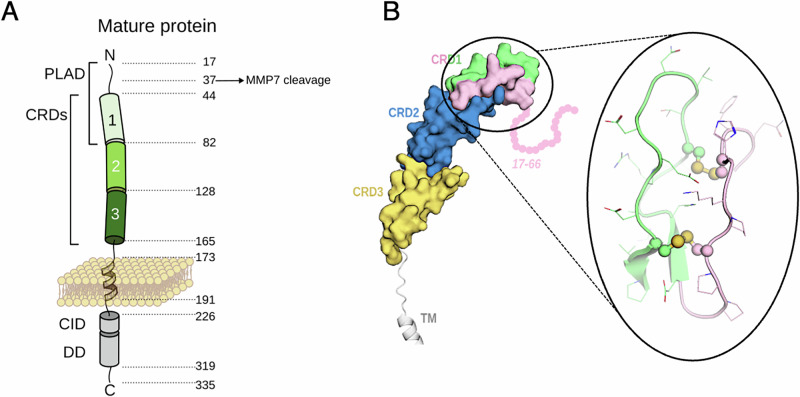
CD95 self-aggregation. **A** Representation of CD95 with in the right, the amino acid numbers (numbering starts from methionine). The MMP7 cleavage site in the N-terminal region of CD95 is indicated [30]. **B** *Left panel.* Solvent accessible surface corresponds to structured domains CRD1, CRD2, and CRD3. The linker and part of the TM domain are shown in cartoons. The flexible sequence corresponds to amino acid residues 17 to 55 and is depicted schematically. In pink, the sequence 17-66, corresponding to mutants Del2 and Del3 [25], spanning the flexible sequence and part of the structured CRD1. The rest of the CRD1 is shaded in green. *Right panel.* Cartoon representation of the CRD1 of CD95 with intra-chain disulfide bonds. In pink, the residues 55-66 that are part of the predicted PLAD. In green, the rest of the CRD1.

Interestingly, to understand the structure of the ectodomain of CD95, only two antibody-bound crystal structures (PDB:3THM and 3TJ3) and, more recently, an AlphaFold prediction (AF-P25445-F1-v4) [24] exist. CD95 contains three cysteine-rich domains (CRDs) in its extracellular region numbered 1 to 3 from the membrane distal N-terminal region (Fig. 1A). All these structural information point out that the structured CRD1 spans from residue 56 to 82, the first 55 amino acid residues being flexible and intrinsically disordered (Fig. 1B). The ectodomain also encompasses two other well-structured CRDs; CRD2 and the upper part of CRD3 are involved in the interaction with CD95L [7]. The PLAD, promoting the TNFR1 and CD95 self-aggregation, was identified in pioneer experiments by Ruberti and Lenardo’s teams [8, 9, 25–27]. For CD95, this domain was initially located in the mature receptor between residues 17 and 82 (numeration starts at methionine in CD95 to match UniProt criteria) [9] (Fig. 1A), a region that spans the disordered segment and the CRD1. Although the PLAD is mandatory for the induction of CD95-mediated signaling pathways [8, 9, 25, 28], it remains to identify the amino acid residues involved in the receptor self-association and besides, to decipher whether the PLAD sequence interacts with itself (homotypic interaction) or with other regions (heterotypic interaction) in CD95. Accordingly, some ALPS type Ia patients can secrete truncated CD95 missense mutants, designated FasDel2 and FasDel3 [25], which have a premature stop codon, a length of 103 and 86 residues, respectively, and span only from amino acids 18 to 66 of CD95 [25]. Both mutants are soluble peptides [25] that abrogate the receptor self-association and prevent the apoptotic signal [9, 27, 29]. Because we demonstrated that metalloprotease MMP-7 cleaves CD95 between amino acids ^36^EL^37^ and does not prevent the pre-ligand association [30], we hypothesize that the minimal PLAD sequence might cover amino acid residues 37 to 66. Based on these findings, we also postulate that the use of a minimal PLAD peptide will compete with this region and prevent CD95 self-association, altering the induction of CD95-mediated signaling pathways involved for instance in the severity of lupus [31, 32]. Such a therapeutic concept has been successfully developed to inhibit the TNFR1-mediated signaling pathways in an arthritis animal model [33].

In addition, the CD95 intracellular region encompasses three domains including a juxtamembrane domain designated the calcium-inducing domain (CID) [31, 32, 34], a death domain (DD) [35], and a C-terminal region (15 last amino acid residues) [36] (Fig. 1A). Recruitment of the adaptor protein Fas-Associated protein with Death Domain (FADD) by the CD95-DD promotes the oligomerization of caspase-8 [37, 38] and the induction of the apoptotic program. Interaction of CD95-CID with phospholipase Cγ1 (PLCγ1) triggers a Ca^2+^ signal promoting cell migration [20, 39–41].

## CD95L structure and functions

CD95L (FasL or CD178) is the ligand of CD95 and is a type II transmembrane protein mainly expressed by immune cells to control the immune response and kill infected and transformed cells [42]. This TNFSF member can also be detected at the surface of endothelial cells during inflammation in auto-immune disorders [31, 32, 43] and cancers [44, 45], in which its function remains to be elucidated. Indeed, while some studies have shown that CD95L expression in endothelial cells prevents leukocyte infiltration [43, 45], we and others demonstrated that this ligand can promote the extravasation of activated T lymphocytes [31, 32] and neutrophils [46], and thereby the accumulation of these immune cells in inflamed tissues. In addition, transmembrane CD95L (mCD95L) can be cleaved by metalloproteases (MMPs) and/or A Disintegrin And Metalloproteases (ADAMs) to release a soluble ligand (sCD95L) that fails to trigger apoptosis [47–51] but induces inflammatory signaling pathways such as NF-kB [52, 53] and PI3K [32, 44]. sCD95L also induces a calcium response *via* the recruitment of the inflammation-promoting phospholipase Cγ1 (PLCγ1) [31, 32, 53, 54]. The identification of the interaction between CD95 and PLCγ1 allowed us to generate a selective inhibitor of this interaction called DB550 (peptidomimetic), which selectively blocks the CD95-mediated PI3K signal [34] and by doing so, alleviates clinical symptoms in a lupus-prone mouse model [34]. The CD95L protomers associate as a compact trimeric complex, in which each monomer adopts a beta sandwich “jelly-roll” structure [55]. These interactions typically result in a 3:3 (receptor:ligand) hexameric structure. Of note, while an homotrimeric CD95L fails to trigger an apoptotic signal, its hexameric counterpart does it, pointing out that CD95L stoichiometry exerts a pivotal function in the quality and magnitude of the implemented CD95 signaling pathways [32, 47, 56]. As aforementioned, the main role of CD95L/CD95 pair is to control the immune response. Accordingly, accumulative evidence indicate that while mCD95L at the plasma membrane of memory T cells can extinguish the immune response by modulating both T [57, 58] and B cell [59] responses, its soluble counterpart stimulates it [51], suggesting that the MMPs and/or ADAMs involved in mCD95L cleavage might represent important modulators of the immune response [60].

## CD95L-independent CD95-mediated cell signaling

CD95 expression by itself seems to control cell signaling pathways through a CD95L-independent mechanism [61–63]. In agreement with this hypothesis, Peter’s group showed that the selective elimination of CD95 gene in ovarian cells abrogates cancer growth [62] in a genetic mouse model of epithelial endometrioid ovarian cancer (i.e., conditional expression of the constitutive active K-rasG12D mutant combined with Pten deletion in ovarian cells [64]). Again, the selective elimination of CD95 in hepatocytes prevents the development of tumors in an inducible hepatocellular carcinoma mouse model (i.e., diethylnitrosamine injection in mice causes hepatocarcinogenesis) [62]. Similarly, the expression of CD95 is also necessary to induce the growth of glioma [63].

Interestingly, the level of CD95 expression is correlated with tumor grade and the severity of the pathology in triple negative breast cancer (TNBC) women [65]. We established that elimination of CD95 expression in TNBC cells releases a KPC2/KPC1-driven NF-kB signaling pathway and the secretion of inflammatory cytokines [61], which might contribute to the induction of a natural killer (NK) cell-driven antitumor response [66]. These findings reveal that CD95 expression in cancer cells can control the NF-kB pathway by promoting a KPC1-dependent ubiquitination of p105 [61], which leads to its partial degradation into p50 [67]. The accumulation of p50 promotes the formation of p50 homodimers, which in turn will compete with the classical NF-kB signal and inhibit it [67]. These findings suggest that elimination of CD95 or disruption of its homodimer complex in certain cancer cells might represent an attractive therapeutic option to prevent a CD95-dependent tumor growth and/or stimulate an inflammatory signal involved in the anti-tumor response of NK cells [66].

## How does CD95 self-associate?

**Fig. 2:**
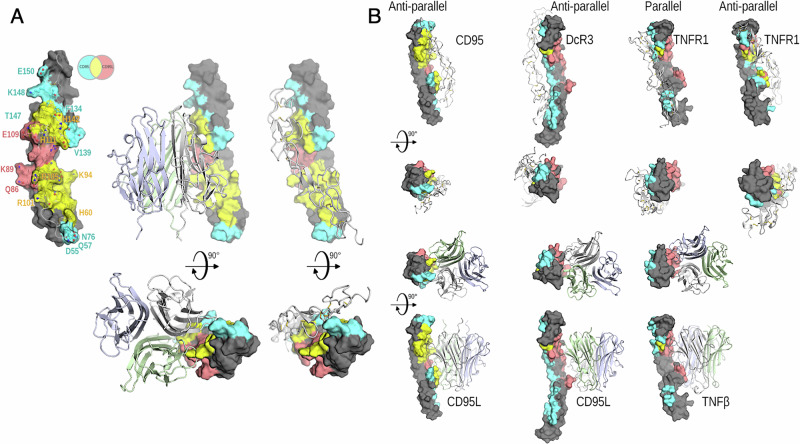
Organization of anti-parallel dimers in TNFR superfamily. **A** CD95 interactions. Left panel. Solvent accessible surface of the AlphaFold model of the CRD domains of CD95. Color shades correspond to the surface involved exclusively in the dimeric (cyan) or in the ligand binding (salmon) interactions, or shared by both (yellow). The same monomer is shown binding the trimeric ligand (center panel) and the other monomer in the anti-parallel dimer (right panel). The bottom representations in the center and right panels are rotated 90 degrees with respect to the upper representation. **B** Dimer of CD95 compared to those of DcR3 and TNFR1. First and second rows show receptor dimeric interactions where only one protomer is shown as solvent accessible surface. From left to right CD95 (AlphaFold Multimer prediction), DcR3 (PDB:4MSV), TNFR1 parallel (PDB:1FT4) and anti-parallel (PDB:1EXT) dimers. Third and fourth rows show the corresponding monomer interacting with its ligand. From left to right, CD95-CD95L (AlphaFold Multimer prediction), DcR3-CD95L (PDB:4MSV), and TNFR1-TNFβ (PDB:1TNR). Unlike the lattice contacts observed in anti-parallel TNFR1 (PDB:1EXT) structure and the predicted CD95 anti-parallel dimer, ligand binding domains in the anti-parallel DcR3 or parallel TNFR1 homodimers (PDB:1TNR) involve opposing faces.

To date, the stoichiometry of CD95 at the plasma membrane remains elusive: while size exclusion chromatography and microscopy experiments indicate that CD95 can self-associate as dimers and trimers [9, 68], high-resolution microscopy methods suggest that CD95 is mainly expressed as a monomer [69]. These discrepancies in the CD95 stoichiometry render difficult to develop a robust model to understand how CD95L interactions can trigger apoptosis or non-apoptotic signaling pathways. In addition, it has been reported that other receptors such as c-Met, can alter the self-association of CD95 at the plasma membrane [70]. Finally, the two X-ray structures of the CD95 extracellular region that were obtained in the presence of agonistic antibodies [71] do not allow us to determine whether and how CD95 can self-associate and how CD95 and CD95L interact. Of note, some TNFRSF members including TNFR1 [72], DR5 [73] and DcR3 [5] have been reported to form ligand-free parallel and anti-parallel dimers, co-existing at the equilibrium [74]. Using AlphaFold-Multimer [24, 75], we evaluated how CD95 might self-aggregate. The molecular modeling (MolMod) approach revealed that this receptor preferentially formed anti-parallel dimers (Fig. 2A, B) and this model also highlighted that the CD95L-binding region was masked by the CD95 interface, suggesting that such CD95 dimers might impair the CD95L binding (Fig. 2A, B) and represent “non-signaling” complexes” [74].

DcR3 is a soluble receptor that interacts with CD95L and neutralizes its activities [76]. The lattice contacts in the crystal structure of DcR3 with CD95L indicates that this soluble receptor forms anti-parallel dimers [2]. Similarly, lattice contacts in the crystals of TNFR1 (PDB:1EXT) reveal the anti-parallel self-association of this receptor and highlight that this dimeric structure buries the TNF binding sites [72]. Based on these findings and our MolMod analyses, we hypothesize that although CD95 has been mainly represented as a parallel dimer/trimer [9], it might actually self-associate in an anti-parallel fashion. In agreement with this hypothesis, a recent study observed that a soluble CD95 fused to *Gaussia princeps luciferase* failed to interact with CD95L, while the same CD95 domain fused to the Fc domain of IgG1 forcing a parallel association, efficiently bound CD95L [77]. Therefore, in solution, an anti-parallel CD95 dimer (“non-signaling” complex) might represent a conformation that masks the domains involved in the CD95L interaction, meaning a basic region including K94, H96, K100, R102, R103, R105, L106 and E109 [78, 79] (Fig. 2A). On the other hand, the anti-parallel DcR3 and TNFR1 dimers still bind CD95L and TNFα, respectively because they exhibit a 90° rotation compared to the CD95 dimer, exposing the ligand binding sites to the solvent (Fig. 2B). Of note, a recent study has identified another “non-signaling” anti-parallel dimerization interaction for TRAILR2 [73], suggesting that the anti-parallel organization might represent a general mechanism of ligand-independent assembly for TNFRSF members.

**Fig. 3:**
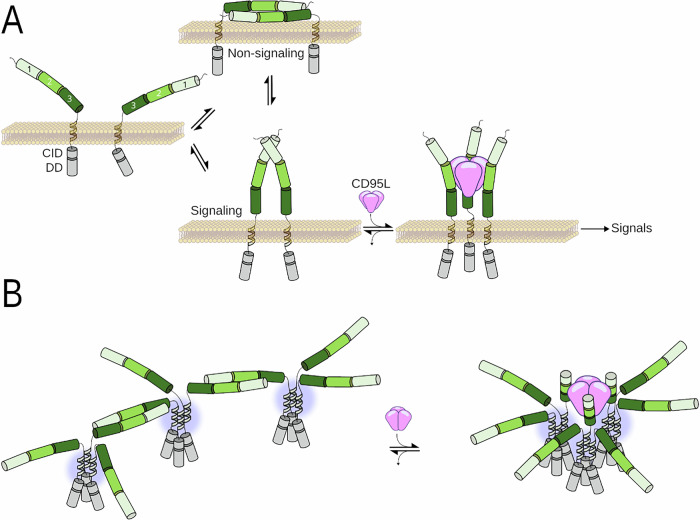
Models of CD95 aggregation at the plasma membrane. **A** CD95 might self-associate after MMP7 cleavage [30] into non-signaling and signaling dimers. In the presence of CD95L, the equilibrium will shift to the signaling dimer and the induction of the apoptotic or non-apoptotic signaling pathways. **B** Predicted stoichiometry and conformation of CD95 protomers at the plasma membrane. To simplify the model, we highlighted in blue the transmembrane domains (TMs). While PLADs (light and dark green cylinders) form dimers, TMs (blue highlight) associate to form trimers [81]. The role of the intracellular domains including CID (calcium-induced domain) [31] and DD (death domain) [98] in the self-aggregation and conformation of CD95 is not depicted in this figure.

Because PLADs of the TNF receptor members self-aggregate with a very weak affinity [80], it is tempting to envision an anti-parallel structure in equilibrium with an unbound and less bended “signaling” receptor (Fig. 3A). In the presence of CD95L, the CD95/CD95L interaction might promote the formation of the CD95 parallel “signaling” form at the expense of the anti-parallel and “non-signaling” structure to foster CD95 aggregation and implement downstream signaling pathways (Fig. 3A). In other words, the addition of CD95L will disrupt the CD95/CD95 “non-signaling” complex to favor the formation of parallel dimers that subsequently expose the CD95L binding site and promote a more favorable receptor-ligand interactions to trigger signaling.

Finally, to elaborate a comprehensive model of CD95 aggregation at the plasma membrane, we have to combine the PLAD-driven self-aggregation with the transmembrane (TM)-induced homo-trimerization [81] (Fig. 3B). Therefore, we envision that without its ligand, the CD95 TMs self-aggregate to form homotrimers [81], while at the same time, PLADs connect two trimers together, creating a “non-signaling” network (Fig. 3B). The binding of the CD95L homotrimer will modify this stoichiometry, disrupting the PLAD interactions to generate a more complex “signaling” network. This “signaling” network remains to be identified but it will i) amplify the aggregation level of CD95 and ii) engender conformation modifications required to release the intracellular death domain and foster FADD binding, an additional step in the CD95 aggregation [82]. Finally, this “signaling” network will initiate a caspase-8-dependent signaling pathway responsible for the recruitment of non-activated CD95 receptors to form larger CD95 aggregates and implement cell signaling [83].

## Role of CD95 signaling pathways in immune responses

### The CD95-mediated immune contraction

The introduction of immunotherapy based on T cells carrying a chimeric antigen receptor (CAR) has profoundly changed the clinical outcomes of cancer patients, especially in hematological malignancies. Multiple myeloma (MM) represents the most common lymphoid malignancy in adults. Several antigens can be targeted on plasma cells, in particular the B-Cell Maturation Antigen (BCMA) and to date, two CAR-T cell products have been authorized since 2021: ciltacabtagene autoleucel (Cilta-cel, Carvyktyi^®^) and idecabtagene vicleucel (Ide-cel, Abecma^®^) [84–86]. Response rates and survival outcomes, beyond the intrinsic characteristics of myeloma cells, appear to be linked to multiple factors including the type of CAR construction and correlated with the expansion capacity, persistence, and functionality of CAR-positive T cells. The CD95/CD95L couple plays a major role in the mechanism of persistence [57]. Memory T cells are involved in the efficacy of the adaptive immune response to pathogens and tumors [58]. Following antigen recognition, memory T cells exhibit a continuum with naive (T_N_), Stem Cell Memory (T_SCM_), Central Memory (T_CM_), Effector Memory (T_EM_) and CD45RA+ Effector Memory (T_EMRA_) similar to exhausted T cells. While T_EM_ cells are committed cells undergoing terminal differentiation (T_EMRA_) after a limited number of divisions, T_CM_ cells constitute a long-lived population through a contingent of T_SCM_. These T_SCM_ cells express characteristics of naive T cells associated with significant amounts of CD122 (IL-2Rb) and CD95 with renewal properties and superior capacity for expansion [87]. Pioneer works from Restifo and Klebanoff’s teams showed that CD95L expression by memory T cells, which remain to be identified in humans, impairs the anti-tumor activity of CD4+ and CD8 + T cells by accelerating the differentiation of T_SCM_ and T_CM_ cells into differentiated T_EM_ cells in adoptive cell therapy [58] and more recently in CAR-T cells-mediated anti-tumor response [57]. This inhibition of the immune response has been designated “precocious differentiation” [57, 58]. It has been recently reported that mCD95L can activate the PI3K signaling pathway in naive T lymphocytes to promote “precocious differentiation” [58]. We postulate that while the expression of CD95L on memory CAR-T cells will accelerate the differentiation/exhaustion of T_SCM_ cells and thus reduce the persistence of CAR-T cells, the release of sCD95L will instead enhance the anti-tumor response.

In agreement with a key role of CD95 in T cell maturation/exhaustion, a genome wide association study (GWAS) combined with a comprehensive immunophenotyping approach of immune cells in twins revealed a link between the proportion of “stem cell memory” cells and variants in a genetic locus encompassing CD95 [88]. Overall, these findings suggest that an interplay exists between the CD95 signal and the modulation of these long-term memory T cells. Accordingly, we wonder whether inhibition of the CD95 dimer/trimer in patients treated with CAR-T cells could represent a therapeutic approach to prevent the “precocious differentiation” of T_SCM_ and increase the anti-tumor response by CAR-T cells.

### sCD95L and inflammation

As aforementioned, mCD95L can be cleaved by MMPs and ADAMs to release a soluble CD95L (sCD95L) [39]. Unlike the membrane-bound CD95L (mCD95L), sCD95L fails to trigger cell death but induces non-apoptotic and pro-inflammatory signaling pathways, which might contribute to the severity of auto-immune and inflammatory disorders including systemic lupus erythematosus (SLE), or Stevens-Johnson syndrome (SJS) and toxic epidermal necrolysis (TEN) (discussed below) and cancers [89]. These data suggest ambivalent physiologic roles for CD95L, with a membrane-bound ligand extinguishing the immune response while its soluble form will stimulate it. From a pathologic standpoint, it will be crucial to exhaustively identify the MMPs and ADAMs responsible for CD95L cleavage in order to assess whether the lack of some of them will engender immunosuppression *via* the accumulation of “precocious differentiation”, while their up-regulation will contribute to the CD95-dependent inflammation contributing to the disease severity in lupus, Stevens-Johnson syndrome (SJS) or toxic epidermal necrolysis (TEN).

## CD95/CD95L therapies

### PC111

Stevens-Johnson Syndrome (SJS) and Toxic Epidermal Necrolysis (TEN) are a continuum of severe skin diseases with necrosis of keratinocytes and disruption of the cutaneous barrier [90], in which soluble CD95L is up-regulated [89]. An antagonist anti-CD95L mAb designated PC111 was developed by PinCell (Milan, Italy) and injected into a humanized mouse model of TEN [89]. It blocked the necrosis extension and prevented the death of the animals suggesting that CD95/CD95L interaction was involved in this pathology. Of note, other antagonist mAbs targeting CD95L have been patented including the NOK family of antibodies (WO1996029350) and our JQ3 (WO2023099578) that could be compared with PC111 in this pathology. The main issue with these antibodies is that they will abrogate both apoptotic and non-apoptotic signaling pathways, rendering difficult to decipher the role of each pathway in the pathology.

### Asunercept

Asunercept, initially APG101, is a decoy receptor consisting in the extracellular region of CD95 fused to the Fc domain of IgG1. This peptide administered to glioblastoma [91], myelodysplastic syndrome (MDS) [92] and COVID-19 patients [93], showed some benefits for the first two pathologies. Although sCD95L is up-regulated in the bronchoalveolar lavage fluid (BALF) of COVID-19 patients who required intubation [94, 95], a phase 2 trial combining asunercept with standard of care (SOC) regimen failed to meet its primary endpoint of time to sustained clinical improvement as compared to SOC treatment alone [93]. Interestingly, sCD95L was also significantly increased in the BALF of patients admitted in intensive care unit (ICU) with severe Influenza A (H1N1) virus infection [94]. Like the aforementioned antagonist mAbs, asunercept will inhibit both apoptotic and non-apoptotic signal and thereby if chronic administering is required, it could affect the positive effect of the apoptotic signal in both the immune homeostasis and the elimination of infected and transformed cells.

### DB550

Based on the identification of the CID-CD95 amino acid residues interacting with PLCγ1, we developed a small peptidomimetic designated DB550, which selectively inhibits the interaction between CD95 and the PLCγ1. DB550 abrogates the Ca^2+^ response in Th17 cells and alleviates clinical symptoms in lupus mice [34, 96, 97]. The advantage of this drug is that it selectively inhibits the Ca^2+^ signal without affecting the CD95-mediated apoptotic pathway [34]. Therefore, administering DB550 to lupus-prone mice, we established the role of the sCD95L-induced non-apoptotic signal in the progression of lupus pathology [34]. We are currently evaluating its therapeutic activity in other auto-immune disorders.

## Conclusion

Most of the attempts to disrupt the CD95/CD95L interactions have focused on targeting the ligand (i.e., asunercept and PC111), putting aside the intrinsic role of CD95 through its ligand independent signaling. This review discusses the CD95L-independent conformation of CD95 at the plasma membrane and suggests that the development of short molecules mimicking PLAD, by disrupting the CD95 homodi- or trimerization might exert an effect similar to the elimination of CD95 in cancer cells. Therefore, along with the inhibition of the CD95L-dependent pro-inflammatory signaling pathways, these structures might also abrogate the CD95L-independent signals observed in TNBC women [61, 66] and thereby, represent original and attractive therapeutics for these patients.
